# Effect of feeding cottonseed meal on some hematological and serum biochemical parameters in broiler birds

**DOI:** 10.14202/vetworld.2016.723-727

**Published:** 2016-07-13

**Authors:** G. Thirumalaisamy, M. R. Purushothaman, P. Vasantha Kumar, P. Selvaraj

**Affiliations:** 1 Division of Animal Nutrition, ICAR – National Dairy Research Institute, Karnal - 132 001, Haryana, India; 2 Department of Animal Nutrition, Veterinary College and Research Institute, Tamil Nadu Veterinary and Animal Sciences University, Namakkal - 637 002, Tamil Nadu, India; 3 Department of Veterinary Physiology, Veterinary College and Research Institute, Tamil Nadu Veterinary and Animal Sciences University, Namakkal - 637 002, Tamil Nadu, India

**Keywords:** broiler, cottonseed meal, hematological and serum parameters, iron supplementation

## Abstract

**Aim:**

The study was undertaken to find out the effect of feeding cottonseed meal (CSM) on performance of hematological and serum biochemical parameters in broiler birds.

**Materials and Methods:**

A 6-week biological trial was carried out with 432-day-old Cobb 400 broiler chicks distributed to nine experimental diets with six replicates, each containing eight chicks. The experimental diets were formulated based on total amino acids (BTAA) or based on digestible amino acids (BDAA) with or without iron supplementation with two levels of CSM (2% and 4%) and control diet based on maize – soybean. The whole blood was subjected to hematological studies. The serum samples were analyzed for protein fractions and lipid profiles.

**Results:**

The packed cell volume (PCV) value, red blood cell (RBC) numbers, and hemoglobin (Hb) were lower in iron unsupplemented CSM BTAA or BDAA diets than the control (33.86-35.54 vs. 36.41%, 2.78-2.87 vs. 2.98 × 10^6^/μl, and 10.30-10.70 vs. 10.88%). Supplementation of iron in CSM diets improved the PCV, RBC numbers, and Hb, and the values were comparable to the control. White blood cell numbers, mean corpuscular volume, mean corpuscular Hb (MCH), and MCH concentration values were comparable to the control. The erythrocyte osmotic fragility (EOF) was poor in birds fed diets containing up to 4% CSM BTAA or CSM BDAA without iron supplementation (32.02-32.57 vs. 28.77%). Supplementation of iron improved the EOF. The serum cholesterol level did not change with or without iron supplementation.

**Conclusion:**

This study suggested that feeding of CSM BTAA or BDAA up to 4% level voiding iron supplementation lowers the hematological parameters, whereas supplementation of iron did not alter serum protein fractions and cholesterol profile; however, it had lowered some hematological parameters, which was rectified by iron supplementation.

## Introduction

In recent years, the cost of conventional protein source for broilers, *viz*., soybean meal has increased by 60%. This increase has resulted in increased cost of broiler meat production. Hence, alternative cheap protein source needs to be evaluated and incorporated in broiler rations to reduce the cost of broiler meat production [1]. The poultry feed industry and nutritional researchers are in search of alternative protein sources which not only supports the highest performance and efficiency but also safeguard the health of the bird and safety of the product.

Cottonseed meal (CSM) has been promising plant protein replacer of the conventional protein meals such as soybean meal and groundnut cake [2]. India is the second largest producer of cottonseed next to China. The production of cottonseed in India was 12.29 MMT in 2013 [3]. For every 1 kg of cotton (*Gossypium* sp.) lint produced, there is an availability of 1.65 kg of cotton seed [4]. The unit (g) price of soybean and CSM protein is 9.3 (42/kg for 450 g of protein) and 5.4 (21/kg for 390 g of protein) paise, respectively. Even a 1% replacement of soybean protein with CSM protein will result in savings of 390/ton of broiler feed.

Modern cottonseed processing industries are equipped to decorticate cotton seed, and hence, the meals obtained are higher in protein and lesser in fiber than the undecorticated CSM. Similarly, the advancement made in the solvent extraction technology has resulted in low oil content [5]. The above two processes in modern cottonseed industry have provided an opportunity to incorporate the decorticated deoiled CSM in the poultry feed.

However, the main problem that has limited its utilization in animal feeding thus far is the presence of gossypol, a toxic polyphenolic compound that naturally found in the pigment glands of the cottonseed [6], and this is present in free and bound forms. The free form is more toxic to monogastric animals. It can negatively affect animal growth, digestive health, and reproduction [7-10]. The free gossypol is higher in direct solvent extracted than mechanical extruded CSM [11]. The cottonseed oil extraction industry employs initially mechanical and subsequently solvent extracted. Hence, the CSM available locally contains low free gossypol due to the destruction of gossypol by heat and pressure [12].

Gossypol readily binds with a free epsilon amino group of lysine during processing, thereby reducing proteolytic action [13]. In addition, gossypol has direct inhibiting action on intestinal enzymes, and it combines with iron thereby reduces hemoglobin (Hb) synthesis and activity of respiratory enzymes. Hence, the use of this CSM is limited. Supplementation of lysine and iron are likely to ameliorate the negative effect of the toxic principle [14]. The objective of the present study was to evaluate the hematological and serum characters of the broiler birds fed CSM based on total amino acids (BTAA) or based on digestible amino acids (BDAAs) with or without iron supplementation.

## Materials and Methods

### Ethical approval

The study was conducted following approved guidelines with the Institutional Animal Ethics Committee and conformed to the “Guidelines for the Care and Use of Animals in Research.”

### Location of study and period

All procedures in the experiment were carried out in the Department of Animal Nutrition, Veterinary College and Research Institute, Namakkal, Tamil Nadu Veterinary and Animal Sciences University (TANUVAS), during the month of November 2014 to January 2015.

### Biological experiment

The experimental broiler pre-starter, starter, and finisher diets were formulated by the inclusion of CSM at varying levels (0%, 2%, and 4% of feed). The various experimental diets are as follows:

- T1 - Standard broiler diet BTAA (control)
- T2 - 2% CSM inclusion BTAA
- T3 - 4% CSM inclusion BTAA
- T4 - 2% CSM inclusion BDAA
- T5 - 4% CSM inclusion BDAA
- T6-T2 with supplementation of ferrous sulfate 1:1 ratio (FeSO_4_: Free gossypol), i.e., 0.4 g of Fe/kg of the diet
- T7-T3 with supplementation of ferrous sulfate 1:1 ratio (FeSO_4_: Free gossypol), i.e., 0.8 g of Fe/kg of the diet
- T8-T4 with supplementation of ferrous sulfate 1:1 ratio (FeSO_4_: Free gossypol), i.e., 0.4 g of Fe/kg of the diet
- T9-T5 with supplementation of ferrous sulfate 1:1 ratio (FeSO_4_: Free gossypol), i.e., 0.8 g of Fe/kg of the diet.

The Treatments 1, 2, and 3 were formulated with the total (reported analytical) amino acids content of the ingredients and the total (reported analytical) amino acid requirement of the birds, i.e., total amino acid indicates both the digested and undigested amino acid component. The Treatments 4 and 5 were formulated with the digestible (reported available) amino acids content of the ingredients and the digestible (reported available) amino acid requirement of the birds, i.e., digestible amino acid indicates the available amino acid component.

The ingredients and nutrient composition of broiler pre-starter, starter, and finisher diets are presented in Table-1. The pre-starter, starter, and finisher diets were fed to birds from 1 to 14, 15 to 28, and 29 to 42 days of age, respectively. The biological experiment was conducted with 432-day-old Cobb 400 broiler chicks. The chicks were wing banded, weighed individually, and assigned randomly to nine experimental diets with six replicates for each diet, and each had eight chicks. Completely randomized design was followed.

**Table-1:** Proximate principles (percent on DM basis), mineral and gossypol content of experiment CSM.

Compositions	Experiment CSM (%)
DM	89.86
Crude protein	39.02
Crude fiber	11.92
Ether extract	3.07
Total ash	7.15
Nitrogen free extract	38.84
Calcium	0.22
Total phosphorus	1.16
Total gossypol	2.62
Free gossypol	0.40

CSM=Cottonseed meal

### Analytical methods

#### Chemical analysis and assay of gossypol

The CSM and the experimental diets were analyzed for proximate principles, calcium, and total phosphorus as per the protocol suggested by AOAC [15]. The total gossypol and free gossypol content of the CSM were analyzed as per AOCS [16,17]. The proximate principles and gossypol content of experiment CSM are presented in Table-2.

**Table-2:** Ingredients (as such basis) and nutrients composition (percent DM) of broiler pre-starter, starter, and finisher diet.

Ingredients (percent)	Pre-starter					Starter					Finisher				
		
	T1	T2	T3	T4	T5	T1	T2	T3	T4	T5	T1	T2	T3	T4	T5
Maize	61.85	61.33	60.82	61.44	60.95	63.67	63.15	62.64	63.27	62.78	61.30	60.91	60.52	60.70	60.33
Soybean meal	33.83	32.26	30.68	32.11	30.50	32.73	31.16	29.58	31.00	29.39	31.37	29.67	27.98	29.69	27.96
Rice bran oil	1.13	1.21	1.30	1.19	1.27	0.81	0.90	0.98	0.87	0.95	4.21	4.28	4.35	4.35	4.42
Calcite	1.96	1.98	2.01	1.98	2.01	2.03	2.05	2.08	2.05	2.08	2.03	2.05	2.07	2.05	2.07
Dicalcium phosphate	0.75	0.72	0.70	0.72	0.70	0.45	0.42	0.39	0.42	0.40	0.47	0.45	0.42	0.45	0.42
Methionine	0.176	0.178	0.181	0.179	0.183	0.140	0.142	0.144	0.143	0.145	0.228	0.230	0.238	0.231	0.239
Lysine	0.229	0.234	0.240	0.296	0.315	0.095	0.100	0.105	0.167	0.187	0.318	0.324	0.330	0.336	0.356
Cottonseed meal	0.00	2.00	4.00	2.00	4.00	0.00	2.00	4.00	2.00	4.00	0.00	2.00	4.00	2.00	4.00
Nutrients* (%)															
Crude protein	22.17	22.08	22.27	22.28	22.25	21.61	21.62	21.54	21.63	21.51	20.32	20.17	20.04	20.27	20.02
Crude fiber	3.94	4.09	4.28	4.10	4.28	3.89	4.00	4.15	4.10	4.17	3.74	3.90	4.24	3.94	4.18
Ether extract	2.83	2.87	2.79	3.30	3.27	4.93	5.08	5.13	5.06	5.10	5.97	6.06	6.15	6.03	5.99
Total ash	6.50	7.68	7.61	6.85	7.12	7.40	6.57	5.96	6.07	5.64	6.25	6.09	6.33	6.90	6.45
Nitrogen free extract	64.56	63.28	63.05	63.48	63.09	62.17	62.74	63.22	63.15	63.59	63.72	63.78	63.24	62.86	63.36
Calcium	1.09	1.10	1.19	1.08	1.06	0.99	1.06	0.97	0.99	0.96	0.96	0.97	0.97	0.99	0.98
Available phosphorus*	0.45	0.45	0.45	0.45	0.45	0.40	0.40	0.40	0.40	0.40	0.40	0.40	0.40	0.40	0.40
Lysine*	1.20	1.20	1.20	1.24	1.26	1.07	1.07	1.07	1.12	1.13	1.16	1.16	1.16	1.17	1.18
Methionine*	0.52	0.52	0.52	0.52	0.52	0.48	0.48	0.48	0.48	0.48	0.54	0.54	0.54	0.54	0.54
Metabolisable energy (Kcal/kg)*	3.00	3.00	3.00	3.00	3.00	3.05	3.05	3.05	3.05	3.05	3.20	3.20	3.20	3.20	3.20

Treatments 6 and 8 are the same as Treatments 2 and 4 with addition of iron sulfate at 0.4 g/kg of feed, and Treatments 7 and 9 are the same as Treatments 3 and 5 with addition of iron sulfate at 0.8 g/kg of feed. Supplied per kg of diet: Vitamin A–16,500 IU, vitamin B_2_–10 mg, vitamin D_3_–3200 IU and vitamin K–2 mg. Supplied per kg of diet: Thiamin–4 mg, pyridoxine–8 mg, cyanocobalamin–40 mcg, vitamin E–40 mg, niacin–60 mg, calcium D pantothenate–40 mg, and folic acid–4 mg. Coccidiostat added at 0.5 g/kg of feed supplied 125 mg of di-nitro-ortho-toluamide. Supplied per kg of diet: Manganese–54 mg, zinc–52 mg, iron–20 mg, iodine–2 mg, copper–2 mg, cobalt–1 mg.

* Calculated value. DM=Dry matter

#### Blood, serum sample collection and analysis

The experimental birds were slaughtered at the end of the trial (42^nd^ day); the blood samples were collected and subjected to hematological studies. After blood collection, the serum samples were harvested and stored at −20°C until further analysis. Total erythrocyte and leukocyte counts were estimated on the same day of blood collection. Hb was estimated by Drabkin and Austin [18] method and packed cell volume (PCV) by microhematocrit centrifugation [19] in the whole blood. Total protein, albumin, globulin, and cholesterol in serum samples were estimated using standard diagnostic kits (Span Diagnostics Ltd., Surat, India). Erythrocyte fragility was measured as percentage hemolysis using 0.65% buffered saline solution [20].

### Statistical analyses

The data generated from the experimental study were subjected to statistical analysis by following the standard procedures of Snedecor and Cochran [21] with the help of IBM SPSS [22] version 20.0 software package. Comparison between groups was made by one-way ANOVA, and the data on hematological and serum parameters were analyzed in repeated measures ANOVA. Results were presented as means and standard error of means. The significance of the difference between means was compared using Duncan’s multiple range test.

## Results and Discussion

### Hematological parameters

The hematological parameters of broilers fed CSM are presented in Table-3. Inclusion of CSM BTAA or BDAA without iron supplementation up to 4% level showed lower PCV value, red blood cell (RBC) numbers, and Hb over the control (33.86-35.54 vs. 36.41%, 2.78-2.87 vs. 2.98 × 10^6^/μl, and 10.30-10.70 vs. 10.88%). This suggests that a negative influence on blood hemopoiesis. Supplementation of iron up to 4% level CSM BTAA or BDAA diet had comparable PCV, RBC numbers, and Hb to the control. In earlier, worker also confirmed that significant reduction in PCV, RBC, and Hb at 30% level of CSM fed birds [23]. Apata [24] suggested that the significant reductions in RBC, RBC, and Hb may be results of factors acting together in an antinutrient containing dietary ingredient to induce inhibition of hemopoiesis, or a combined toxic factor-induced RBC hemolysis leading to an increase in plasma volume. This work was consistent with observations of Reddy and Salunkhe [25] and Kannan *et al*. [26], who stated that dietary antinutrients form a complex with dietary irons at intestinal or tissue level which, in turn, results in the diminished oxygen carrying capacity of blood. The antinutrient iron complex, in turn, reduces the amount of iron required for the functioning and regeneration of RBC, which is reflected in Hb.

**Table-3:** Effect of feeding CSM on hematological parameters in broilers.

Treatments	PCV (%)	RBC (10^6^/µl)	WBC (10^3^/µl)	Hb (g/dl)	MCV (fl)	MCH (pg)	MCHC (%)	EOF (%)
T1	35.93^b^±0.28	2.95^c^±0.05	3.23±0.07	10.88^c^±0.15	122.21±1.95	36.96±0.56	30.27±0.34	28.77^a^±0.60
T2	33.98^a^±0.15	2.81^ab^±0.03	3.29±0.03	10.47^ab^±0.06	121.24±1.14	37.34±0.36	30.81±0.25	32.27^c^±0.66
T3	33.68^a^±0.40	2.76^a^±0.03	3.26±0.05	10.30^a^±0.07	122.25±1.70	37.38±0.36	30.63±0.45	32.57^c^±1.17
T4	34.29^a^±0.16	2.82^ab^±0.06	3.33±0.04	10.50^ab^±0.07	121.91±1.64	37.32±0.48	30.63±0.28	32.02^bc^±1.13
T5	34.07^a^±0.15	2.80^ab^±0.04	3.32±0.04	10.43^ab^±0.13	121.84±1.09	37.29±0.60	30.59±0.28	32.41^c^±0.66
T6	35.54^b^±0.23	2.88^abc^±0.03	3.30±0.08	10.66^bc^±0.10	123.61±1.12	37.06±0.36	30.01±0.40	30.33^abc^±1.19
T7	35.40^b^±0.26	2.84^abc^±0.04	3.26±0.07	10.62^abc^±0.12	124.77±1.55	37.40±0.44	30.00±0.33	30.96^abc^±1.07
T8	35.86^b^±0.14	2.92^bc^±0.03	3.16±0.05	10.70^bc^±0.11	122.94±0.87	36.69±0.45	29.85±0.38	29.12^ab^±0.99
T9	35.31^b^±0.21	2.90^bc^±0.04	3.11±0.04	10.63^abc^±0.11	121.99±1.23	36.71±0.47	30.09±0.18	29.67^abc^±0.88

Each value is the mean of 12 observations. Mean with at least one common superscript in a column do not differ significantly (p>0.05). MCV=Mean corpuscular volume, PCV=Packed cell volume, RBC=Red blood cell, Hb=Hemoglobin, WBC=White blood cell, MCH=Mean corpuscular hemoglobin, MCHC=Mean corpuscular hemoglobin concentration, EOF=Erythrocyte osmotic fragility, CSM=Cottonseed meal

White blood cell numbers, mean corpuscular volume, mean corpuscular Hb (MCH), and MCH concentration (MCHC) values were not significantly alter whether diet is formulated CSM BTAA or BDAA with or without iron supplementation when compared to the control.

The erythrocyte osmotic fragility (EOF) was increased when diet included at 4% CSM BTAA or CSM BDAA without iron supplementation compared to the control (31.98-32.89 vs. 28.77%). However, supplementation of iron up to 4% level in CSM BTAA or BDAA had observed comparable EOF to the control diet. Osmotic fragility of erythrocytes measures the ability of erythrocytes to resist osmotic stresses. Reyes *et al*. [27] observed that gossypol binds strongly to lipid bilayers and induces an electrical conductance that is accompanied by an increase in proton permeability. This affects the fluidity of membranes. In the present study, EOF was affected by treatment suggesting alteration of membrane integrity due to iron addition to the diet which may also explain the changes in Hb and MCHC values among treatments. Iron in the diet, on the other hand, was able to eliminate some of the negative effects of gossypol in CSM.

### Serum parameters

The results of the biochemical estimation of serum protein fractions are presented in Table-4. The serum protein fractions in terms of total protein, albumin, globulin, and albumin globulin ratio did not significantly influence between the all dietary treatments.

**Table-4:** Effect of feeding CSM on serum protein fractions in broilers.

Treatments	Total protein (g/dl)	Albumin (g/dl)	Globulin (g/dl)	A/G ratio
T1	4.12±0.07	1.79±0.03	2.34±0.07	0.77±0.03
T2	4.10±0.08	1.71±0.03	2.39±0.07	0.72±0.02
T3	4.08±0.07	1.79±0.03	2.29±0.09	0.80±0.04
T4	4.10±0.07	1.70±0.02	2.40±0.05	0.71±0.02
T5	3.93±0.09	1.74±0.03	2.19±0.09	0.81±0.04
T6	3.87±0.08	1.71±0.03	2.16±0.09	0.81±0.04
T7	4.01±0.07	1.75±0.03	2.26±0.07	0.78±0.03
T8	4.03±0.07	1.73±0.02	2.31±0.06	0.76±0.02
T9	3.90±0.06	1.76±0.02	2.14±0.07	0.84±0.03

Each value is the mean of 12 observations. CSM=Cottonseed meal

Serum cholesterol values are presented in Table-5. The serum cholesterol in terms of total cholesterol HDL, LDL, and triglycerides did not statistically significant between the dietary treatments. The present study clearly indicates that all the diets supported normal balance between the anabolism and catabolism of body proteins. It concurs with previous workers reported inclusion of CSM at 10% in broilers [28], 20% in lambs [26], and complete replacement of low gossypol CSM in laying hens [29] were not affecting the serum protein fractions and cholesterol values.

**Table-5:** Effect of feeding CSM on serum cholesterol in broilers.

Treatments	Total cholesterol (mg/dl)	HDL (mg/dl)	LDL (mg/dl)	Triglycerides (mg/dl)
T1	204.58±1.80	98.16±1.09	88.15±2.59	91.37±1.71
T2	205.52±1.96	97.31±0.79	89.75±2.08	92.29±1.43
T3	202.64±2.11	99.18±1.07	84.97±2.41	92.43±1.43
T4	201.24±1.78	95.06±0.72	87.53±1.75	93.27±1.06
T5	205.32±1.12	95.65±1.39	89.84±2.77	94.15±0.83
T6	205.24±1.25	98.20±1.16	88.14±1.29	94.52±1.12
T7	205.94±2.43	98.37±0.73	88.91±2.30	93.36±0.84
T8	202.72±2.11	99.22±1.51	84.85±2.94	93.22±1.07
T9	200.86±1.56	97.55±1.24	84.54±1.64	93.83±1.20

Each value is the mean of 12 observations. HDL=High-density lipoprotein, LDL=Low-density lipoprotein, CSM=Cottonseed meal

## Conclusions

CSM BTAA or BDAA up to 4% level without iron supplementation had lowered hematological parameters, whereas supplementation of iron in CSM BTAA or BDAA had enhanced the hematological parameters. The serum protein and cholesterol parameters did not significantly influence when the diet formulated CSM BTAA or BDAA up to 4% level.

## Authors’ Contributions

This study was a part of M.V.S.c. thesis of the first author GT, who carried out the research under the guidance of Professor and Head MRP. PVK and PS helped during trial and laboratory analyses. The article was drafted by GT and MRP. The revision was made by GT and MRP. All authors have read and approved the final version of the manuscript.
